# Supplementary material for: Segmentation of time series in up- and down-trends using the epsilon-tau procedure with application to USD/JPY foreign exchange market data
Source: PLoS One. 2020 Sep 18;15(9):e0239494. doi: 10.1371/journal.pone.0239494 (PMC7500655; doi:10.1371/journal.pone.0239494)
Supplement: S1 Appendix — (PDF) [file pone.0239494.s001.pdf]

Segmentation of time series in up- and down-trends using the epsilon-tau procedure with application to USD/JPY foreign exchange market data

Arthur Matsuo Yamashita Rios de Sousa, Hideki Takayasu, Misako Takayasu

Supporting information

S1 Appendix. Trend length and trend amplitude marginal probability distributions from the epsilon-tau procedure for random walks.

The epsilon-tau procedure presented in the main text – considering time constant patience level τ and tolerance level for the up-trend case $\varepsilon = \max_{\{m+1 \le t' \le t\}} x_{t'} - x_m$, where m is the reference point – imposes restrictions on the sequence of values x_t of a time series that can form an up-trend (analogous for down-trend).

The tolerance level ε restricts the values x_t of the up-trend $[m+1, m+\ell]$ to be always above the reference value x_m:

$$x_t > x_m, \forall t \in [m+1, m+\ell]. \tag{1}$$

The patience level τ requires that for all points t before the end of the trend $m+\ell$ there is at least one point t' in the window $[t+1, \min(t+\tau, m+\ell)]$ with at least the same value of t (otherwise the end of the trend would be t because the time between consecutive maximum values would have reached the patience level):

$$\exists t' \in [t+1, \min(t+\tau, m+\ell)] : x_{t'} \ge x_t, \forall t \in [m+1, m+\ell-1]. \tag{2}$$

For points beyond the end of the trend $m+\ell$, we must have either one of the following set of restrictions arising from the stop conditions:

(a) value of time series reaches tolerance level ε:

$$\begin{cases} x_m < x_t < x_{m+\ell}, \forall t \in [m+\ell+1, m+\ell+\mu-1]; \\ x_{m+\ell+\mu} \le x_m, \end{cases} \tag{3}$$

where $1 \le \mu \le \tau$.

(b) time between consecutive maxima reaches patience level τ:

$$x_m < x_t < x_{m+\ell}, \forall t \in [m+\ell+1, m+\ell+\tau]. \tag{4}$$

Observe that from the above restrictions we have that the first increment $\xi_{m+1} = x_{m+1} - x_m$ of an up-trend is always positive and the first increment $\xi_{m+\ell+1} = x_{m+\ell+1} - x_{m+\ell}$ beyond the end of an up-trend is always negative.

Using the constraints for up-trends, we derive the trend length and trend amplitude marginal probability distributions for the random walk:

$$x_t = x_{t-1} + \xi_t, \tag{5}$$

where the independent and identically distributed increments ξ_t can take value $+1$ with probability p, -1 with probability q, or 0 with probability $r = 1 - p - q$. In the derivation, we take the reference point $m = 0$ to simplify the notation.

Trend length marginal probability distribution

For patience level $\tau = 1$, the restrictions on the increments of the random walk translate as:

$$\begin{cases} \xi_1 = +1; \\ \xi_t = +1 \text{ or } \xi_t = 0, \forall t \in [2, \ell]; \\ \xi_{\ell+1} = -1. \end{cases} \tag{6}$$

And thus the probability of an up-trend with length ℓ for $\tau = 1$ is:

$$\begin{aligned} P(up, \ell; \tau = 1) &= P(\xi_1 = +1) \left\{ \prod_{t=2}^{\ell} \Big[P(\xi_t = +1) + P(\xi_t = 0) \Big] \right\} P(\xi_{\ell+1} = -1) \\ &= p(p + r)^{\ell-1} q. \end{aligned} \tag{7}$$

For patience level $\tau = 2$, we have two cases according to the trend amplitude a:

(i) Trend amplitude $a = 1$:

$$\begin{cases} \xi_1 = +1; \\ \xi_t = 0, \forall t \in [2, \ell]; \\ \xi_{\ell+1} = -1. \end{cases} \tag{8}$$

The probability of an up-trend with length ℓ and amplitude $a = 1$ is:

$$\begin{aligned} P(up, \ell, a = 1; \tau = 2) &= P(\xi_1 = +1) \left[\prod_{t=2}^{\ell} P(\xi_t = 0) \right] P(\xi_{\ell+1} = -1) \\ &= pr^{\ell-1} q. \end{aligned} \tag{9}$$

(ii) Trend amplitude $a \geq 2$:

$$\begin{cases} \xi_1 = +1; \\ \xi_t = 0, \forall t \in [2, \nu + 1]; \\ \xi_{\nu+2} = +1; \\ \begin{cases} \xi_{t+1} = +1 \text{ or } \xi_{t+1} = 0 \text{ or } \xi_{t+1} = -1, \text{ if } \xi_t = +1 \\ \xi_{t+1} = +1 \text{ or } \xi_{t+1} = 0 \text{ or } \xi_{t+1} = -1, \text{ if } \xi_t = 0 \\ \xi_{t+1} = +1, \text{ if } \xi_t = -1 \end{cases} , \forall t \in [\nu + 3, \ell - 1]; \\ \xi_\ell = +1 \text{ or } \xi_\ell = 0; \\ \xi_{\ell+1} = -1; \\ \xi_{\ell+2} = 0 \text{ or } \xi_{\ell+2} = -1, \end{cases} \tag{10}$$

where ν, $0 \leq \nu \leq \ell - 2$, is the number of zero increments between the first positive increment ξ_1 and the next positive increment $\xi_{\nu+2}$.

We obtain the probability of an up-trend with length ℓ, amplitude $a \geq 2$ and number of initial zero increments ν by considering a Markov process in the increments ξ_t with transition matrix $\mathbf{T}_{(\xi)}$:

$$\begin{aligned} \mathbf{T}_{(\xi)} &= \begin{bmatrix} P(\xi_{t+1} = +1 \mid \xi_t = +1) & P(\xi_{t+1} = +1 \mid \xi_t = 0) & P(\xi_{t+1} = +1 \mid \xi_t = -1) \\ P(\xi_{t+1} = 0 \mid \xi_t = +1) & P(\xi_{t+1} = 0 \mid \xi_t = 0) & P(\xi_{t+1} = 0 \mid \xi_t = -1) \\ P(\xi_{t+1} = -1 \mid \xi_t = +1) & P(\xi_{t+1} = -1 \mid \xi_t = 0) & P(\xi_{t+1} = -1 \mid \xi_t = -1) \end{bmatrix} \\ &= \begin{bmatrix} p & p & p \\ r & r & 0 \\ q & q & 0 \end{bmatrix} \end{aligned} \tag{11}$$

$$
\begin{aligned}
P(up,\ell,a\geq 2,\nu;\tau=2) &= \Big[P(\xi_{\ell+2}=0)+P(\xi_{\ell+2}=-1)\Big]P(\xi_{\ell+1}=-1)\\
&\quad\times\begin{bmatrix}1 & 1 & 0\end{bmatrix}\mathbf{T}_{(\xi)}^{\ell-\nu-2}\begin{bmatrix}1\\0\\0\end{bmatrix}P(\xi_{\nu+2}=+1)\left[\prod_{t=2}^{\nu+1}P(\xi_t=0)\right]P(\xi_1=+1)\\
&= \frac{p^2q(r+q)}{\sqrt{(p+q)^2+4pq}}\\
&\quad\times\left[-\left(\frac{2r}{p+r-\sqrt{(p+q)^2+4pq}}\right)^{\nu}\left(\frac{p+r-\sqrt{(p+q)^2+4pq}}{2}\right)^{\ell-1}\right.\\
&\quad\left.+\left(\frac{2r}{p+r+\sqrt{(p+q)^2+4pq}}\right)^{\nu}\left(\frac{p+r+\sqrt{(p+q)^2+4pq}}{2}\right)^{\ell-1}\right].
\end{aligned}
\tag{12}
$$

Therefore, the probability of an up-trend with length ℓ for patience level $\tau=2$ is:

$$
\begin{aligned}
P(up,\ell;\tau=2) &= P(up,\ell,a=1;\tau=2)+\sum_{\nu=0}^{\ell-2}P(up,\ell,a\geq 2,\nu;\tau=2)\\
&= pr^{\ell-1}q+\frac{p^2q(r+q)}{\sqrt{(p+q)^2+4pq}}\\
&\quad\times\left\{-\left(\frac{p+r-\sqrt{(p+q)^2+4pq}}{p-r-\sqrt{(p+q)^2+4pq}}\right)\left[\left(\frac{p+r-\sqrt{(p+q)^2+4pq}}{2}\right)^{\ell-1}-r^{\ell-1}\right]\right.\\
&\quad\left.+\left(\frac{p+r+\sqrt{(p+q)^2+4pq}}{p-r+\sqrt{(p+q)^2+4pq}}\right)\left[\left(\frac{p+r+\sqrt{(p+q)^2+4pq}}{2}\right)^{\ell-1}-r^{\ell-1}\right]\right\}.
\end{aligned}
\tag{13}
$$

It would be possible to derive the probability distributions for patience level $\tau\geq 3$ using higher order Markov chains, but the computation becomes involved by such method and we do not develop it here.

Trend amplitude marginal probability distribution

In order to derive the probability of an up-trend with amplitude a for arbitrary patience level τ, we use the combinatorial approach schematized in Fig 1-a: we represent an up-trend of amplitude a by a positive increments intercalated by boxes b_h, $1\leq h\leq a$, to be filled with sequences of increments with zero sum, i.e., not contributing to the trend amplitude, while respecting the restrictions due to the patience and tolerance levels, and a final box b_{a+1} to be filled with a sequence of increments indicating the stop of the epsilon-tau procedure (either due to the tolerance level or to the patience level). Fig 1-b shows the representation of a set of minimal sequences of increments with zero sum having length j (≥ 1) and maximum depth k (≥ 0), where the value of the initial position is only repeated in the end of the sequence – any sequence of increments respecting those limits can occupy the shaded gray area. An indefinite number of such minimal sequences can be inserted in each box b_h, $1\leq h\leq a$, provided that $j\leq\tau$ (so that the patience level is not reached) and the depth k of the sequence does not reach the reference value x_0 of the up-trend (tolerance level is not reached). Fig 1-c represents a set of sequences indicating the stop of the epsilon-tau procedure when the tolerance level ε is reached; the initial position of the sequence cannot be revisited and its length j must be at most τ and its depth k must be equal to the trend amplitude a, reaching the reference value x_0 of the up-trend only in the end of the sequence. Fig 1-d represents a sequence indicating the stop of the epsilon-tau procedure when the patience level τ is reached; the initial position of the sequence cannot be revisited either and its length j must be equal to τ and its maximum depth k must be $a-1$ (not reaching the tolerance level).

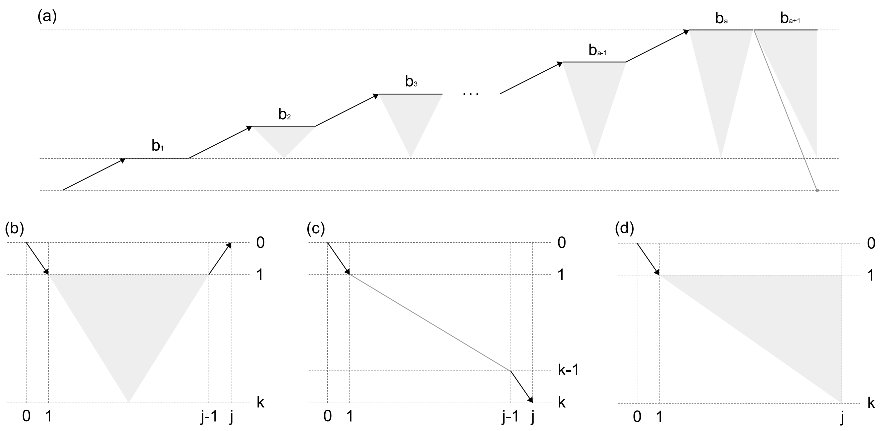

Figure 1: **Combinatorial approach to derive the up-trend amplitude probability distribution for random walk.** (a) Representation of an up-trend of amplitude a, where boxes b_h, $1 \leq h \leq a$, are to be filled with sequences of increments with zero sum while respecting the restrictions due to the patience and tolerance levels and box b_{a+1} is to be filled with a sequence indicating the stop of the epsilon-tau procedure. (b) Representation of a set of minimal sequences of increments with zero sum to be inserted in a box b_h, $1 \leq h \leq a$. (c) Representation of a set of sequences indicating the stop of the epsilon-tau procedure when the tolerance level ε is reached to be inserted in the box b_{a+1}. (d) Representation of a set of sequences indicating the stop of the epsilon-tau procedure when the patience level τ is reached to be inserted in the box b_{a+1}.

We compute the probabilities of each mentioned set of sequences by utilizing a Markov process in the positions y_t (and not in the increments ξ_t, as done for the trend length). Note the different notation y_t for the position in the sequence of each set being studied and not the position x_t in the whole up-trend. The transition matrix $\mathbf{T}_k$ of order k in this case reads as:

$$\mathbf{T}_k = \begin{bmatrix} P(y_t = 1 \mid y_{t-1} = 1) & P(y_t = 1 \mid y_{t-1} = 2) & P(y_t = 1 \mid y_{t-1} = 3) & \cdots & P(y_t = 1 \mid y_{t-1} = k) \\ P(y_t = 2 \mid y_{t-1} = 1) & P(y_t = 2 \mid y_{t-1} = 2) & P(y_t = 2 \mid y_{t-1} = 3) & \cdots & P(y_t = 2 \mid y_{t-1} = k) \\ P(y_t = 3 \mid y_{t-1} = 1) & P(y_t = 3 \mid y_{t-1} = 2) & P(y_t = 3 \mid y_{t-1} = 3) & \cdots & P(y_t = 3 \mid y_{t-1} = k) \\ \vdots & \vdots & \vdots & \ddots & \vdots \\ P(y_t = k \mid y_{t-1} = 1) & P(y_t = k \mid y_{t-1} = 2) & P(y_t = k \mid y_{t-1} = 3) & \cdots & P(y_t = k \mid y_{t-1} = k) \end{bmatrix}$$
$$= \begin{bmatrix} r & p & 0 & \cdots & 0 \\ q & r & p & \cdots & 0 \\ 0 & q & r & \cdots & 0 \\ \vdots & \vdots & \vdots & \ddots & \vdots \\ 0 & 0 & 0 & \cdots & r \end{bmatrix}_{k \times k} \tag{14}$$

(i) Set z_{jk} of minimal sequences of length j and maximum depth k with zero sum :

- Case $j \geq 2, k = 0$: there is no minimal sequence in this set z_{jk} because for length $j \geq 2$ it is necessary at least one negative and one positive increments – and thus a depth $k \geq 1$ – to have a sequence with zero sum. Then:

$$P(z_{jk}) = 0, \text{ if } j \geq 2, k = 0. \tag{15}$$

- Case $j = 1, k \geq 0$: the only possible sequence in this set z_{jk} is the one formed by a single zero increment with initial position $y_0 = 0$ and final position $y_1 = 0$. The probability is:

$$P(z_{jk}) = P(y_1 = 0 \mid y_0 = 0) = r, \text{ if } j = 1, k \geq 0. \tag{16}$$

- Case $j \geq 2, k \geq 1$: represented in Fig 1-b, sequences in this set z_{jk} have negative increment from initial position $y_0 = 0$, positive increment to final position $y_j = 0$ and all intermediate positions in between $y_t = 1$ (otherwise the sequence would not be minimal) and $y_t = k$ (the maximum depth). The probability of of this set is given by (using results on powers of tridiagonal toeplitz matrices – reference [28] of the main text):

$$
\begin{aligned}
P(z_{jk}) &= P(y_j = 0 \mid y_{j-1} = 1) \begin{bmatrix} 1 & 0 & 0 & \ldots & 0 \end{bmatrix}_{1\times k} \mathbf{T}_k^{j-2} \begin{bmatrix} 1 \\ 0 \\ 0 \\ \vdots \\ 0 \end{bmatrix}_{k\times 1} P(y_1 = 1 | y_0 = 0) \\
&= \frac{2pq}{k+1} \sum_{u=1}^{k} \lambda_{\frac{u}{k+1}}^{j-2} \sin^2\left(\frac{u\pi}{k+1}\right), \text{ if } j \geq 2, k \geq 1,
\end{aligned}
\tag{17}
$$

where $\lambda_{\frac{u}{k+1}} = r + 2\sqrt{pq}\cos\left(\frac{u\pi}{k+1}\right)$.

(ii) Set $s_{jk}^{(\varepsilon)}$ of sequences of length j and depth k indicating the stop of the procedure due to the tolerance level ε:

- Case $j \geq 1, k = 0$: there is no sequence in this set $s_{jk}^{(\varepsilon)}$ because sequences indicating the stop of the epsilon-tau procedure starts with a negative increment and, thus, $k \geq 1$. Then:

$$P(s_{jk}^{(\varepsilon)}) = 0, \text{ if } j \geq 1, k = 0. \tag{18}$$

- Case $j = 1, k \geq 2$: a sequence with depth $k \geq 2$ must have length $j \geq 2$. Then:

$$P(s_{jk}^{(\varepsilon)}) = 0, \text{ if } j = 1, k \geq 2. \tag{19}$$

- Case $j \geq 2, k = 1$: because sequences in $s_{jk}^{(\varepsilon)}$ start and ends with negative increment, there is no sequence in this set. The probability is:

$$P(s_{jk}^{(\varepsilon)}) = 0, \text{ if } j \geq 2, k = 1. \tag{20}$$

- Case $j = 1, k = 1$: the first increment of a sequence indicating the stop of the epsilon-tau procedure must be negative, which already satisfies the conditions of this set $s_{jk}^{(\varepsilon)}$. Thus:

$$P(s_{jk}^{(\varepsilon)}) = P(y_1 = 1 \mid y_0 = 0) = q, \text{ if } j = 1, k = 1. \tag{21}$$

- Case $j \geq 2, k \geq 2$: represented in Fig 1-c, sequences in this set $s_{jk}^{(\varepsilon)}$ start with a negative increment from initial position $y_0 = 0$ and end with a negative increment to final position $y_j = k$; all intermediate positions must be in between $y_t = 1$ (because the initial position cannot be revisited) and $y_t = k - 1$ (because depth k is only reached in the final position). Then, the probability of this set is:

$$
\begin{aligned}
P(s_{jk}^{(\varepsilon)}) &= P(y_j = k \mid y_{j-1} = k-1) \\
&\times \begin{bmatrix} 0 & 0 & 0 & \ldots & 1 \end{bmatrix}_{1\times(k-1)} \mathbf{T}_{k-1}^{j-2} \begin{bmatrix} 1 \\ 0 \\ 0 \\ \vdots \\ 0 \end{bmatrix}_{(k-1)\times 1} P(y_1 = 1 | y_0 = 0) \\
&= \frac{2q^2}{k}\left(\frac{q}{p}\right)^{\frac{k-2}{2}} \sum_{u=1}^{k-1} \lambda_{\frac{u}{k}}^{j-2} \sin\left(\frac{u\pi}{k}\right) \sin\left(\frac{(k-1)u\pi}{k}\right), \text{ if } j \geq 2, k \geq 2.
\end{aligned}
\tag{22}
$$

(iii) Set $s_{jk}^{(\tau)}$ of sequences of length j and maximum depth k indicating the stop of the procedure due to the patience level τ:

- Case $j \geq 1, k = 0$: there is no sequence in this set $s_{jk}^{(\tau)}$ since sequences indicating the stop of the epsilon-tau procedure starts with a negative increment ($k \geq 1$). Then:

$$P(s_{jk}^{(\tau)}) = 0, \text{ if } j \geq 1, k = 0. \tag{23}$$

- Case $j \geq 1, k \geq 1$: represented in Fig 1-d, sequences in this set $s_{jk}^{(\tau)}$ start with a negative increment from initial position $y_0 = 0$ and all other positions must be in between $y_t = 1$ (because the initial position cannot be revisited) and $y_t = k$ (the maximum depth). Then, the probability of this set is:

$$\begin{aligned} P(s_{jk}^{(\tau)}) &= \begin{bmatrix} 1 & 1 & 1 & \dots & 1 \end{bmatrix}_{1\times k} \mathbf{T}_k^{j-1} \begin{bmatrix} 1 \\ 0 \\ 0 \\ \vdots \\ 0 \end{bmatrix}_{k\times 1} \qquad P(y_1 = 1 | y_0 = 0) \\ &= \sum_{u=1}^{k} \frac{2q}{k+1} \left(\frac{q}{p}\right)^{\frac{u-1}{2}} \sum_{v=1}^{k} \lambda_{\frac{v}{k+1}}^{j-1} \sin\left(\frac{uv\pi}{k+1}\right) \sin\left(\frac{v\pi}{k+1}\right), \text{ if } j \geq 1, k \geq 1. \end{aligned} \tag{24}$$

We can now write an expression for the probability of an up-trend with amplitude a for arbitrary patience level τ. In each box b_h, $1 \leq h \leq a$, we can insert any number of sequences from the set union $\bigcup_{j=1}^{\tau} z_{j(h-1)}$ and in box b_{a+1} we place a single sequence from the set union $\left[\bigcup_{j=1}^{\tau} s_{ja}^{(\varepsilon)}\right] \cup s_{\tau(a-1)}^{(\tau)}$. Therefore, we have:

$$\begin{aligned} P(up, a; \tau) &= P(\xi = +1)\left\{\sum_{u=0}^{\infty}\left[P\left(\bigcup_{j=1}^{\tau} z_{j0}\right)\right]^u\right\} P(\xi = +1)\left\{\sum_{u=0}^{\infty}\left[P\left(\bigcup_{j=1}^{\tau} z_{j1}\right)\right]^u\right\} \\ &\times P(\xi = +1)\left\{\sum_{u=0}^{\infty}\left[P\left(\bigcup_{j=1}^{\tau} z_{j2}\right)\right]^u\right\} \cdots P(\xi = +1)\left\{\sum_{u=0}^{\infty}\left[P\left(\bigcup_{j=1}^{\tau} z_{j(a-1)}\right)\right]^u\right\} \\ &\times P\left(\left[\bigcup_{j=1}^{\tau} s_{ja}^{(\varepsilon)}\right] \cup s_{\tau(a-1)}^{(\tau)}\right) \\ &= P(\xi = +1)\left\{\sum_{u=0}^{\infty}\left[\sum_{j=1}^{\tau} P(z_{j0})\right]^u\right\} P(\xi = +1)\left\{\sum_{u=0}^{\infty}\left[\sum_{j=1}^{\tau} P(z_{j1})\right]^u\right\} \\ &\times P(\xi = +1)\left\{\sum_{u=0}^{\infty}\left[\sum_{j=1}^{\tau} P(z_{j2})\right]^u\right\} \cdots P(\xi = +1)\left\{\sum_{u=0}^{\infty}\left[\sum_{j=1}^{\tau} P(z_{j(a-1)})\right]^u\right\} \\ &\times \left[\sum_{j=1}^{\tau} P(s_{ja}^{(\varepsilon)}) + P(s_{\tau(a-1)}^{(\tau)})\right] \\ &= \frac{p}{1 - \sum_{j=1}^{\tau} P(z_{j0})} \frac{p}{1 - \sum_{j=1}^{\tau} P(z_{j1})} \cdots \frac{p}{1 - \sum_{j=1}^{\tau} P(z_{j(a-1)})} \left[\sum_{j=1}^{\tau} P(s_{ja}^{(\varepsilon)}) + P(s_{\tau(a-1)}^{(\tau)})\right] \\ &= \left[\prod_{k=0}^{a-1} \frac{p}{1 - \sum_{j=1}^{\tau} P(z_{jk})}\right] \left[\sum_{j=1}^{\tau} P(s_{ja}^{(\varepsilon)}) + P(s_{\tau(a-1)}^{(\tau)})\right]. \end{aligned} \tag{25}$$
