# Supplementary material for: Segmentation of time series in up- and down-trends using the epsilon-tau procedure with application to USD/JPY foreign exchange market data
Source: PLoS One. 2020 Sep 18;15(9):e0239494. doi: 10.1371/journal.pone.0239494 (PMC7500655; doi:10.1371/journal.pone.0239494)
Supplement: Attachment — Submitted filename: Response_to_Reviewers.docx [file pone.0239494.s002.docx]

-------------------------------------------------------
Response to Editor -- PONE-S-20-19486
-------------------------------------------------------

The authors thank the editor for carrying out the review process of the manuscript PONE-S-20-19486. Please find below our comments on his concerns.

-----

“*Reviewers find this paper quite interesting and they suggest its publication after minor revision. However, one of them has major concerns that I share. I agree that the state of the art should consider some recently published papers. Apart from the suggested by the reviewers I would suggest also to revise doi.org/10.1371/journal.pone.0188814.”*

**Our answer:** We are pleased to know that the reviewers found our manuscript interesting. We agree with the editor and reviewers that our introduction would be more complete if we presented recent works on more general time series segmentation in the financial context. We added reference 10 to 17, including the ones suggested by the editor and reviewers, and added to the text: (line 8) “Especially in the context of finance, various techniques to segment time series by identifying periods with similar behavior or by finding switching points were developed [10–17].”

“*My major concern (see comments of reviewer 1 and 2) is the limitation of this study to the case of the Brexit Referendum and the USDJPY currency pair. I would like to be sure that this is not an important limitation in the application of the results obtained in this paper.”*

**Our answer:** We thank the editor for presenting his concerns. We first clarify that the application of the method was not limited to the Brexit Referendum period; in fact, we analyzed a four years period from 2015 to 2018, including indeed the Brexit Referendum as a major event but not the only one (for example, see Table 2, that includes the China’s Black Monday, the US election and several announcements from the Bank of Japan and Federal Reserve). We started presenting the segmentation of the week of the Brexit Referendum in section “Up- and down-trends in financial time series” only as an illustration (it would be impractical and of little value to show the segmentation results for all the weeks); the statistical analyses were performed for the whole period from 2015 to 2018. To make it clear, we added: (line 152) “We use the week when the Brexit Referendum took place to illustrate the segmentation of financial time series.” and (line 166) “trends obtained from the segmentation of the financial time series for the whole four years period”.

Regarding the use of the USD/JPY currency pair only, we do not see it as an important limitation of our work. We do not aim, for instance, at a detailed study of the effects of the Brexit Referendum in different markets. The objective of our manuscript is to introduce a new time series segmentation method (the epsilon-tau procedure) and use it to investigate the internal structure of trends, in particular the identification abnormal trend shapes that do not occur in the random case. The segmentation method itself and the tool for finding abnormal trend shapes are not limited to the financial context (for example, we used the segmentation procedure in discrete random walks – section “Up- and down-trends in random walks”). By analyzing the USD/JPY pair, we provided evidence of the suitability of our methods for the investigation of financial time series.

“*Please explain the rationale for the development of your tool in light of recent research in this area, clearly indicating which problem with existing tools you are addressing.*

*Please clearly report at the beginning of your methods or results section which were the key performance measures used to establish the validity and utility of your method.*

*Please also report clearly which statistical analysis was used to establish robustness of performance measures. (…) To demonstrate the performance of the method, we would expect comparisons to be drawn between existing state-of-the-art methods.”*

**Our answer:** The proposed epsilon-tau procedure is in the line of research of methods to segment time series in up- and down trends. It is a direct generalization of state-of-the-art epsilon-drawdown method, that only uses a tolerance level ε to define an up- or down-trend. We extend it by using simultaneously a tolerance level ε and a patience level τ, allowing more control and flexibility to the time series segmentation. This explanation is explicit in the text: (line 22) “In this work, we unify and extend those previous ideas and propose the epsilon-tau procedure, that simultaneously makes use of a tolerance level ε and a patience level τ to determine up- and down-trends in time series.” Being the epsilon-drawdown method our main reference, we do draw several comparisons between it and our epsilon-tau procedure: (line 57) “The epsilon-drawdown method used in [23-25] can be regarded as a particular instance of the epsilon-tau procedure with infinite patience level τ → ∞”; (line 89) “Such asymptotic exponential behavior also occurs when applying the epsilon-tau procedure to the considered random walk”; (line 197) “The distributions corresponding to the original market data decay slower than the exponential ones of the random cases (...), which is in accordance with the results using the epsilon-drawdown method in financial markets [24].” In especial, the comparison of the distributions shows that our method reproduces results of state-of-the-art epsilon-drawdown method, corroborating its validity, but in addition it allow us to study trends in different time scales (microtrends, macrotrends…) by adjusting the patience level τ, which exemplifies its utility (e.g., (line 161) “with small τ emphasizing the microtrends and large τ, the trends regarded as macrotrends (for the one week time frame)”).

Another contribution of our work is the study of the internal structure of the trends, an aspect underexplored in the cited previous works. In order to highlight this contribution we added: (line 31) “We pay special attention to the internal structure of the trends, performing a systematic investigation of trend shapes that occur in the market time series and introducing an approach based on the Fisher's exact test to select abnormal shapes that are rarely produced when the data is randomized.”

Regarding the clustering of similar trend shapes, we compute the standard deviation for all points in the normalized trends in a cluster to assess the similarity of the trend shapes ((line 241) “we show the normalized trends in the cluster by plotting all normalized trends in gray, the average in black and the standard deviation in pink”). And to select trend shapes that do not occur in the randomized data, we use the Fisher's exact to evaluate the significance of our results ((line 254) “we apply the Fisher's exact test to check if the actual proportion of trends from original and from randomized data in a cluster is incompatible with the proportion of the total trends of each category supposing the null hypothesis of randomly selecting trends to compose the cluster.”).

Therefore, we believe that all required information is in the present version of the manuscript.

---------------------------------------------------------------
Response to Reviewer #1 -- PONE-S-20-19486
---------------------------------------------------------------

The authors thank the reviewer for carefully reading the manuscript PONE-S-20-19486 and for the comments that contributed to its improvement. Please find below our answers to the questions and criticisms included in the report.

-----

“*Overall and interesting and well written paper.”*

**Our answer:** We are glad that the reviewer found our paper interesting.

“*It is very very rare to see a paper that does not compare to some strawman. You need to compare to something, or make a strong case as to why you do not need to.”*

**Our answer:** The proposed epsilon-tau is a generalization of the epsilon-drawdown method, that only uses a tolerance level ε to define an up- or down-trend. We extend it by using simultaneously a tolerance level ε and a patience level τ (see (line 22) “In this work, we unify and extend those previous ideas and propose the epsilon-tau procedure, that simultaneously makes use of a tolerance level ε and a patience level τ to determine up- and down-trends in time series.”). Being the epsilon-drawdown method our main reference, we do draw several comparisons between it and our procedure: (line 57) “The epsilon-drawdown method used in [23-25] can be regarded as a particular instance of the epsilon-tau procedure with infinite patience level τ → ∞”; (line 89) “Such asymptotic exponential behavior also occurs when applying the epsilon-tau procedure to the considered random walk”; (line 197) “The distributions corresponding to the original market data decay slower than the exponential ones of the random cases (...), which is in accordance with the results using the epsilon-drawdown method in financial markets [24].” In especial, the comparison of the distributions shows that our method reproduces results of state-of-the-art epsilon-drawdown method, corroborating its validity, but in addition it allow us to study trends in different time scales (microtrends, macrotrends…) by adjusting the patience level τ, which exemplifies its utility (e.g., (line 161) “with small τ emphasizing the microtrends and large τ, the trends regarded as macrotrends (for the one week time frame)”).

Also note that we study the trends internal structures, an aspect underexplored in the cited previous works. In order to highlight this contribution we added: (line 31) “We pay special attention to the internal structure of the trends, performing a systematic investigation of trend shapes that occur in the market time series and introducing an approach based on the Fisher's exact test to select abnormal shapes that are rarely produced when the data is randomized.”

“‘*we define the distance between trends as the Euclidean distance between the corresponding normalized trends using the sampled points’ Please expand, this is not clear to me. Would two time series with identical slope have a zero distance?”*

**Our answer:** We agree with the reviewer that this point needs clarification. We rewrote: (line 218) “For the distance between trends, we normalize the trends by setting unit length and unit amplitude (that is, we rescale the original trend horizontally by its length and vertically by its absolute amplitude) and sample a fixed number of points from the normalized trend at fixed positions (we take 100 equidistant points); we define the distance between trends as the Euclidean distance between the vectors formed by the sampled points from the corresponding normalized trends. For example, if we have two perfectly linear trends, the distance between them is zero independent of their lengths or amplitudes, confirming that they have exactly the same shape; on the other hand, we can have trends with same length and amplitude but with distance greater than zero because they have distinct shapes.”

Answering the specific question of the reviewer, we first note that the trends are not necessarily linear (nor even approximately linear). If we define slope as amplitude divided length, then trends with identical slope can have distance > 0 if their shapes (“the relative position of all points (or a sample of points) of the trend”) are different (see added text above).

“*Could you get similar results with a much similar way? For example, if you did PLA segmentation (your ref [2]) would that give you similar results to fig 4 etc”*

**Our answer:** Most segmentation methods (with adequate parameters values) would yield a figure resembling the specific Fig 4. However, it would be only a visual similarity and the extracted trends would be essentially different. Using the example of the Piecewise Linear Approximation (PLA), the trends would be approximately linear, while in our method there is not a predetermined trend shape. This fact is of great importance to our clustering analysis: if all trends are approximately linear, all trend shapes are similar and there would be no meaning in the study of trend shapes. In order to make it explicit in the text, we added: (line 53) “We remark that the epsilon-tau procedure does not require a predetermined functional form by which trends are approximated, as it happens, for instance, in piecewise linear methods, where trends are approximated by straight lines [2]; this is an important feature that allow us to explore the diversity of possible trend shapes.”

“‘*For 17 of its 28 down-trends, we could associated the sharp mid-quote drops with exceptional events in the studied period (...) and with announcements from the Federal Reserve and Bank of Japan’ This strikes me as a little post-hoc”*

**Our answer:** The comment of the reviewer is indeed relevant. In order to make the study completely transparent, we added: (line 282) “By searching for the date and time of the trends in specialized media (…)”. Moreover, to add a layer of caution, we included: (line 302) “Note, however, that no associated events were found for the remaining 11 trends in cluster D and there are probably other major events associated with different trend shapes, reminding us that this is still an incipient study and that the relationship between trend shapes and market events needs to be further investigated”.

“‘*The raw data used in this study was purchased from EBS Service Company Limited (...). I will let this slide, but normally I do not accept papers until the code and data is all freely available.”*

**Our answer:** We understand the position of the reviewer, but we are restricted by contract and cannot share the data (that is the drawback of analyzing this kind of data). However, we restate that we had no special access privileges and any interested researcher is able to purchase the same data we analyzed.

---------------------------------------------------------------
Response to Reviewer #2 -- PONE-S-20-19486
---------------------------------------------------------------

The authors thank the reviewer for carefully reading the manuscript PONE-S-20-19486 and for the comments that contributed to its improvement. Please find below our answers to the questions and suggestions included in the report.

*-----*

“*In this paper the authors described a time series segmentation method called the epsilon-tau method. According to the authors, this method was first suggested by Johansen and Sornette in 2010 in passing but never fully developed. In this method, a time series segment no longer than a patience tau is called an uptrend if the ending value exceeds the starting value by greater than a tolerance epsilon. By varying epsilon and tau, and applying the method to the one-second time series data of the US dollar-Japanese yen exchange rate between 2015 and 2018, the authors examined a group of 28 highly significant downtrends and found that 17 of them can be attributed to known events.
This is an impressive piece of work. Overall, it is well written, and I recommend for it to accepted for publication, after the authors address my following minor concerns:”*

**Our answer:** We are glad with the overall positive evaluation of our manuscript. The reviewer provides a fair summary of our work. We just comment that the method suggested by Johansen and Sornette in 2010 only considered a single parameter similar the patience level τ, while in our work we take both patience level τ and tolerance level ε simultaneously, representing then an extension of the suggested idea and resulting in more controllable and flexible method.

*“(1) I feel that the description of the epsilon-tau procedure needs to be more detailed, because what is written is somewhat confusing. For example, if a segment of the time series has x(t_end) - x(t_start) > epsilon, but at time t_mid, x(t_mid) - x(t_start) < epsilon, do we still consider this one segment (from t_start to t_end), or more than one segment (t_start to t_mid, and t_mid to t_end)?”*

**Our answer:** We thank the reviewer for pointing out the confusing part. One of the conditions to stop the procedure is that a time series value reaches the tolerance level ε; if the tolerance level is reached at t_mid, then the corresponding trend is already determined and the procedure is restarted to search for the next trend (that is, we would have more than one trend). Note that the point in which the time series reaches the tolerance level is not the end of the trend but just a flag to indicate the stop of the procedure. In the example of the reviewer, if the tolerance level is reached at t_mid, the trend end is at some t_beforemid < t_mid, where the value of t_beforemid is a maximum in the considered segment (for the up-trend case). To clarify this point, we added: (line 50) “Observe that the trend ends in m+l and not in t^∗^; the point t^∗^ indicates the stop of the procedure (…)”.

*“(2) After understanding that the segmentation method is based on identifying robust local trends in the time series, I feel that it is similar in spirit to the DNA walk method by Peng et al. (Peng, C.K., Buldyrev, S.V., Goldberger, A.L., Havlin, S., Sciortino, F., Simons, M. and Stanley, H.E., 1992. Fractal landscape analysis of DNA walks. Physica A: Statistical Mechanics and its Applications, 191(1-4), pp.25-29), and should therefore cite this and similar papers.“*

**Our answer:** The reviewer suggests a paper performing an analysis that depends on the identification of trends in the time series using the called min-max procedure. That is indeed an interesting paper to be cited in our introduction when we exemplify the segmentation of time series in diverse fields. We thank the reviewer for the suggestion.

---------------------------------------------------------------
Response to Reviewer #3 -- PONE-S-20-19486
---------------------------------------------------------------

The authors thank the reviewer for carefully reading the manuscript PONE-S-20-19486 and for the comments that contributed to its improvement. Please find below our answers to the questions, criticisms and suggestions included in the report.

*-----*

“*The authors from the paper ‘segmentation of time series in up- and down-trends using the epsilon-tau procedure’ present a research work on time series in financial markets (in particular the currency exchange market) where time segmentation and trend analysis is of interest with regard to historical market data research. The document is correctly written, and the mathematical model is well presented, where I do not find major issues. Thus, the paper is ready for publication after revision, which involves:”*

**Our answer:** We are pleased with the overall positive assessment of our paper by the reviewer. We expect that the answers we provided to the comments are satisfactory and the reviewer is convinced that the current version of our manuscript is suitable for publication.

*“1.- State of the art: At the introduction, the authors promptly jump into the topic of time series segmentation in the field of finance, namely financial markets. Here it would be good to introduce past and present research from the field. A good summary on market dynamics can be found at (…) should be considered as these papers depict the time evolution on financial markets from a scope where dynamic periods are identified where markets, such as the currency exchange one, display physical phases as arrested crystal or glass states, clustered and random ones where prices diffuse. In these works, such states relate to phase transitions and risk management in a similar way in which the authors from the paper propose a time segmentation in market signals, in particular, the currency exchange one.”*

**Our answer:** We agree with the reviewer that our introduction would be more complete if we presented recent works on more general time series segmentation in the financial context and thank her/him for the suggestions. We added reference 10 to 17, including the two papers suggested by the reviewer, and added to the text: (line 8) “Especially in the context of finance, various techniques to segment time series by identifying periods with similar behavior or by finding switching points were developed [10–17]”. We chose to not include reference McCauley2009 because we think it is too broad for the main purpose of our manuscript, which is to present the segmentation method and study the internal structure of trends.

*“2.- Authors consider events for time series analysis. Here they focus on the Brexit Referendum from June 2016. This is a good period of analysis as short and middle-term fluctuations where significant in many markets due to the events in the UK. However, I wonder:
a. Why do authors focus only on the USDJPY currency pair? As they mention in the paper is the GBPUSD is the pair that displayed the largest fluctuations, decreasing down to historical magnitudes. Also, in this case, it would be worthwhile to consider the dynamics of other significant pairs such as the EURGBP or the GBPJPY. I don’t understand why the focus on the USDJPY only and will acknowledge I authors can explain this point.*

*b. Authors select the Brexit referendum and the USDJPY in their study. However, this is a single event. I will acknowledge if trend analysis and segmentation where discussed in terms of other events that influenced financial markets and in particular the Currency Exchange Market. For example, Trump’s victory on 2016, 09/11 and maybe most important to this study, the EURUSD correction in magnitude indirectly forced by the ECB with the Quantitative Easing purchase program. Here, the BCE directed a decrease of the EURUSD pair as the EUR was too high vs. the USD at the second half of the Great Recession, which helped in European exports and liquidity in the system. I encourage the authors to check for this data in the time period between late 2014 and late 2016 as a long-term negative trend is clearly observed. I think that adding such contents in the analysis, even if latter on the paper deeps in the USDJPY, would help in broadening the scope in which the proposed methodology can be considered and applied.”*

**Our answer:** We thank the reviewer for presenting his concerns. We first clarify that the application of the method was not limited to the Brexit Referendum period; in fact, we analyzed a four years period from 2015 to 2018, including indeed the Brexit Referendum as a major event but not the only one (for example, see Table 2, that includes the China’s Black Monday, the US election and several announcements from the Bank of Japan and Federal Reserve). We started presenting the segmentation of the week of the Brexit Referendum in section “Up- and down-trends in financial time series” only as an illustration (it would be impractical and of little value to show the segmentation results for all the weeks); the statistical analyses were performed for the whole period from 2015 to 2018. To make it clear, we added: (line 152) “We use the week when the Brexit Referendum took place to illustrate the segmentation of financial time series.” and (line 166) “trends obtained from the segmentation of the financial time series for the whole four years period”.

Regarding the use of the USD/JPY currency pair only, we do not see it as an important limitation of our work. We do not aim, for instance, at a detailed study of the effects of the Brexit Referendum in different markets. The objective of our manuscript is to introduce a new time series segmentation method (the epsilon-tau procedure) and use it to investigate the internal structure of trends, in particular the identification abnormal trend shapes that do not occur in the random case. The segmentation method itself and the tool for finding abnormal trend shapes are not limited to the financial context, but by analyzing the USD/JPY pair we provided evidence of the suitability of our methods for the investigation of financial time series.

*“3.- Authors choose key magnitudes such as a patience level \tau=7200 seconds from figure 7(a), or the one in figure 11 \tau=1800 seconds. I might have missed it, but I do not fully understand why the selection of such magnitudes. What is particular to them?”*

**Our answer:** The patience level τ = 7200 was solely chosen for Fig 7-a in order to allow the visualization of the trends in the one week time scale (we added to the text: (line 158) “In this example of financial time series segmentation, we use patience level τ = 7200 (2h) for better visualization of the trends in the one week time frame”) – if τ was too small, we would only see intercalated red and blue points and Fig 7-a would lose its purpose. For the statistical analyses we selected patience levels τ = 60 (1min), 600 (10min) and 1800 (30min). There is nothing particular to those specific values, but they refer to different trend time scales (see (line 161) “with small τ emphasizing the microtrends and large τ, the trends regarded as macrotrends (for the one week time frame)” and (line 169) “τ = 60 (1min) (highlighting microtrends), τ = 600 (10min) (intermediate case) and τ = 1800 (30min) (highlighting macrotrends)”). If τ was too big we would have only few long trends per week. But (line 73) “the choice of its value is (...) connected to the characteristics of the observer and her/his intentions; for example, if the aim is the development of real-time applications, a small τ is more suitable, but for historical analysis, larger τ values can provide valuable information.”

*“4.- Probabilities and cumulative probabilities with trend amplitude or trend length clearly display an exponential character (as plotted in log-lin graphs), or similar. Here it would be helpful to quantify such data. It looks like that a stretched or expanded exponential could be a good fit (~exp(-(t/tc)^p), with tc a critical variable), where parameters such as p are significant of the type of fluctuations found in the currency and the time interval (for example random or correlated ones).*

*5.- Finally, it would be interesting to know about the scaling in the model. As exposed before, authors choose a particular currency and a particular time-frame. However, it would be interesting to know if the model scales if a broader or smaller sample is chosen (thus, another event). This is significant as literature states that events that induce strong volatility in different markets (such as Brexit, 9/11, etc) have a limited time extension after which markets stabilize. Thus, such fluctuations are high but improbable events, that disappear in long term due to market self-correcting. Even more, if scaling occurs, I would appreciate some comments related to Efficient Markets and the Efficient Market Hypothesis.“*

**Our answer:** The reviewer indicates very interesting research directions using our method to analyze market data (as she/he also does in comment 2). However, we believe that the proposed analyses are out of the intended scope of present manuscript. As explained in the answer for comment 2: “The objective of our manuscript is to introduce a new time series segmentation method (the epsilon-tau procedure) and use it to investigate the internal structure of trends, in particular the identification abnormal trend shapes that do not occur in the random case.” We highlighted the focus of our analysis by adding: (line 31) “We pay special attention to the internal structure of the trends, performing a systematic investigation of trend shapes that occur in the market time series and introducing an approach based on the Fisher's exact test to select abnormal shapes that are rarely produced when the data is randomized.” The analyses proposed by the reviewer are in the same spirit of the ones in our reference [24] (Filimonov V, Sornette D. Power law scaling and Dragon-Kings in distributions of intraday financial drawdowns. Chaos Solitons Fractals. 2015;74:27-45), in which the authors provide a detailed study of the distributions and discuss the scaling using their epsilon-drawdown method. We agree that it would be interesting to systematically compare those results with the ones from our epsilon-tau procedure and to further investigate the role of the patience level in the distributions, but in our view such study deserves a separated paper.
