# Supplementary material for: Segmentation of time series in up- and down-trends using the epsilon-tau procedure with application to USD/JPY foreign exchange market data
Source: PLoS One. 2020 Sep 18;15(9):e0239494. doi: 10.1371/journal.pone.0239494 (PMC7500655; doi:10.1371/journal.pone.0239494)
Supplement: Attachment — Submitted filename: Response_to_Reviewers.docx [file pone.0239494.s003.docx]

---------------------------------------------------------------
Response to Reviewer #3 -- PONE-S-20-19486
---------------------------------------------------------------

The authors thank the reviewer for her/his comments on the revision of the manuscript PONE-S-20-19486. Please find below our reply.

*-----*

“*The authors either applied appropriate modifications to the paper or provided valid explanations in reply to questions and concerns. However, there is one issue that should be considered. As stated in the previous review, the manuscript is focused on the USDJPY currency pair. Here, despite authors claim that \epsilon - \tau segmentation can be employed in other currencies (or markets). I wonder about the output in cumulative probability (either depending on trend length or amplitude) in the case of markets where volatility can be much remarkable such as the case of the EURUSD, or the opposite, low volatility markets such as the EURCHF.*

*In the case of the EURUSD and in the last one-two months (June-July 2020), the pair has displayed important trends as result of the health and economic impact of the covid19 disease at different scales. For example, the last Fed announcement was responsible for a highly volatile situation, with trends displaying significant amplitudes at high frequencies. However, at long times the pair displayed remarkable up-trends at least till about two to three weeks ago. I wonder about the probability and cumulative output in the case of the EURUSD, mainly due to its short-term volatility. For example, at trend lengths large enough, could cumulative probabilities (or probabilities) resolve echoes due to high frequency activity? Note that trends are characterized by channels, in practice defined by parallel or divergent Bollinger Bands. As well I wonder in the case of low volatility markets.*

*Therefore, and in lack of any analysis other than the one performed on the USDJPY, it would be clarifying to the contents of the manuscript that the title indicates that the study is performed on the USDJPY currency pair. Besides this change, I find that the last version submitted is of interest to PLoS ONE. If the authors consider mentioning the USDJPY pair in the title as focus of the study, the paper is ready for acceptance.*

**Our answer:** The reviewer presents pertinent points to be explored using our method and they should definitely be investigated in a future work focused on the comparison between different currency pairs. We agree with the reviewer that we should state the scope of our application in the title of the manuscript and modified it to “Segmentation of time series in up- and down-trends using the epsilon-tau procedure with application to USD/JPY foreign exchange market data”. We thank the reviewer once more for her/his positive evaluation and valuable comments.
